# The Association between Seasonal Variation in Vitamin D, Postural Sway, and Falls Risk: An Observational Cohort Study

**DOI:** 10.1155/2013/751310

**Published:** 2013-10-07

**Authors:** Marie-Louise Bird, Keith D. Hill, Iain Robertson, Madeleine J. Ball, Jane K. Pittaway, Andrew D. Williams

**Affiliations:** ^1^School of Human Life Sciences, University of Tasmania, Launceston, TAS 7250, Australia; ^2^School of Physiotherapy, Curtin University, Perth, WA 6845, Australia

## Abstract

*Introduction*. Low serum vitamin D levels are associated with increased postural sway. Vitamin D varies seasonally. This study investigates whether postural sway varies seasonally and is associated with serum vitamin D and falls. *Methods*. In a longitudinal observational study, eighty-eight independently mobile community-dwelling older adults (69.7 ± 7.6 years) were evaluated on five occasions over one year, measuring postural sway (force platform), vitamin D levels, fall incidence, and causes and adverse outcomes. Mixed-methods Poisson regression was used to determine associations between measures. *Results*. Postural sway did not vary over the year. Vitamin D levels varied seasonally (*P* < 0.001), peaking in summer. Incidence of falls (*P* = 0.01) and injurious falls (*P* = 0.02) were lower in spring, with the highest fall rate at the end of autumn. Postural sway was not related to vitamin D (*P* = 0.87) or fall rates, but it was associated with fall injuries (IRR 1.59 (CI 1.14 to 2.24, *P* = 0.007). *Conclusions*. Postural sway remained stable across the year while vitamin D varied seasonally. Participants with high values for postural sway demonstrated higher rates of injurious falls. This study provides important evidence for clinicians and researchers providing interventions measuring balance outcomes across seasons.

## 1. Introduction

Balance impairment is an important fall-risk factor [1], and increases in range of postural sway in the mediolateral direction in older adults are associated with increased fall-risk and rates [2]. Postural sway has been shown in older adults to be strongly related to other measures of balance [3]. Multivariate analysis reveals serum vitamin D levels as an independent variable associated with postural sway [4]. In individuals with suboptimal levels of vitamin D, balance and strength improve after supplementation [5], in particular postural sway [6]. Epidemiological studies have shown that vitamin D levels show seasonal variation [7, 8]. Lowest levels of serum vitamin D are recorded towards the end of winter, approximately four weeks after the shortest day of the year [8]. Overall, vitamin D supplementation did not reduce rate of falls (RaR 1.00, 95% CI 0.90 to 1.11; seven trials; 9324 participants) or risk of falling (RR 0.96, 95% CI 0.89 to 1.03; 13 trials; 26, 747 participants) but may do so in people with lower vitamin D levels before treatment [9]. Older adults are at risk for lower levels of serum vitamin D because of age-related changes in UVB absorption and skin capacity to synthesize vitamin D, reduction in activation in the kidneys, and reduced expression of vitamin D receptors in tissues [5].

There are many factors affecting fall-risk for older individuals, and although these may be different for inside and outside falls [10], strength and balance remain two important physical fall-risk factors. A recently published overview of the literature supports an assertion that age-related changes in postural reactions may be related to vitamin D status-mediated through either central nervous system integration or antigravity muscles as the effectors in postural responses [11]. Despite changes in vitamin D across the seasons [8], muscle strength in the quadriceps muscles has been shown to remain stable [12]. The relationship of postural sway to any potential increased winter fall rate and decreased levels of vitamin D has not been investigated.

The winter season sees an increase in injuries from falls and in the number of accidental deaths from falls [13]. Fracture rates from falls in older adults also increase at the end of the winter season [8], following two to eight weeks after the nadir in serum vitamin D levels. Research into seasonal variation in fall rates, however, has produced disparate results. Some studies report an increased rate of falls, for both inside and outside falls [13, 14]; however, significant seasonal variation in fall rates was not found in a three-year study [8] while, in a second study, seasonal variation in fall rates was reported in women but not in men [15].

Coincident static balance changes with any potential increased fall rates in winter have not been previously reported. The data presented here forms part of a larger study, from which two other papers with the same clinical trial registration have been published [12, 16]. The aim of this study was to determine differences in static balance (postural sway), vitamin D, incidence of falls, and type of fall serially at the end of each season over a 12-month period, in older community-living adults. A secondary aim was to determine associations between seasonal variations in these variables. We hypothesised that postural sway, falls, and vitamin D would show a seasonal variation and that there would be an inverse relationship between vitamin D and the other variables.

## 2. Materials and Methods

### 2.1. Study Design

At the end of consecutive seasons, static balance, vitamin D status, and fall rate were measured within a longitudinal study design. No intervention was implemented by study researchers, so that the study could identify natural variations that occur over the seasons. Data was collected over a three-week period in each season from end of spring 2009 to the end of spring 2010, with collection of data timed to coincide with expected peaks and troughs in serum vitamin D levels [8] in Australia at latitude 41 degrees south (Tasmania). After each assessment, participants were given an appointment for the next collection block in three months' time.

### 2.2. Participants

Independently living community-dwelling adults aged between sixty and eighty-five years were recruited through local print media and community clubs. All participants were able to ambulate independently. Exclusion criteria included recent or current acute medical conditions or an uncontrolled chronic condition. Daily intake of oral supplementation of vitamin D of greater than 800 international Units was also an exclusion criterion. Participants were also excluded if they had a history of neurological disease and were withdrawn if they suffered a medical condition while participating in the study that would impact on their ability to perform the physical tests. Liver and kidney disease both impact vitamin D metabolism, and any potential participants with either of these conditions were excluded.

A priori sample size calculation was based on a previous study reporting mediolateral sway range in a sample of community-dwelling older adults [17], and this indicated a minimum requirement of 81 completed participants (minimum effect size 2.5 mm sway; standard deviation (SD) 8 mm; power 0.8, alpha 0.05). Ninety-eight participants were recruited with the anticipation of a 15% drop-out rate. This repeated measures cohort study was designed to be able to detect differences in postural sway. This project received ethical approval from the Human Research Ethics Committee (Tasmania) Network (H0010561).

### 2.3. Outcome Measures

Postural sway range in the medio-lateral sway direction was measured using a force platform (AMTI Accugait PJB 101, Massachusetts, USA) for thirty seconds under conditions of eyes open and closed, as well as with the additional challenge of using a 6.5 cm foam cushion (eyes open and eyes closed) (Airex Elite Balance Pad AG, Switzerland). Participants were asked to remain stationary with their arms by their sides and look straight ahead while standing on the force platform. No practice trials were given. Foot position (bare feet) was standardised with heels 4 cm apart using marked placement for the feet to ensure repeatability between testing occasions. Maximum excursion range during the 30 seconds was recorded.

Venous blood samples were collected and clotted, then centrifuged (1610 relative centrifugal force) for 15 minutes. The separated sera were aliquoted and stored until analysis at −80°C. Samples for each participant were analysed in the same batch to reduce interassay variation. Serum concentration of 25-hydroxy vitamin D was measured by direct, competitive chemiluminescent immunoassay in a commercial accredited laboratory, using LIAISON method (DiaSorin Inc., Stillwater, MN, USA).

Participants received an individually coded calendar for 12 months of the study on which to report any falls and associated details by date, including information regarding the location and cause of the fall. Information about the type of fall, any injuries that resulted, and if medical attention was sought was recorded. Diaries were returned each month via a free return paid envelope. This study utilised the WHO definition of a fall as “an event which results in a person coming to rest inadvertently on the ground or other lower level” [18]. 

### 2.4. Statistical Analysis

Annual cyclic trends were investigated by fitting a sine wave formula to data for postural sway and vitamin D, with the amplitude of the seasonal variation (in percentage change) and the annual mean values were estimated using repeated measures nonlinear regression, adjusted for age and gender. For secondary analysis the three monthly data were interpolated linearly to estimate intermediate values to correspond with the observed fall incident data. Mixed-methods Poisson regression was used to determine associations between falls, fall injuries, and postural balance and vitamin D. The association between postural balance and vitamin D and season was estimated using mixed-methods linear regression, adjusted for age, gender, and strength. For comparison, seasonal data for falls was grouped into autumn and winter and compared to spring and summer [14].

Physical activity using the CHAMPS questionnaire and muscle strength using the physiological profile assessment tools were recorded, and this information has been published elsewhere [12] but forms an integral part of the meta-analysis of this paper.

## 3. Results

### 3.1. Participants

Data from eighty-eight participants (70% females) are included in the final analysis. Five people did not attend appointments, and five people could not complete testing because of medical events. The participants had a mean (SD) age of 69.2 (6.5) years and body mass index 27.4 (3.9) kg · m^−2^. All participants were living in their own homes independently, with only 10% being sole occupants. Common chronic controlled health conditions included cardiovascular disease (39%) and arthritis (14%). Twenty-six percent of the participants reported the use of more than 4 medications.

### 3.2. Outcomes

Postural sway data for the five time points are provided in Table 1. All four balance measures had the highest sway scores (poorest balance) at the first end of spring measurement. All other seasonal measures were significantly different from this first time point (all *P* < 0.05), but no subsequent significant difference was seen after any other seasonal measures, indicating a lack of seasonal variation in this outcome (*P* > 0.05). No associations between postural sway and vitamin D were observed (all *P* > 0.05). Increased postural sway was associated with fall injuries (IRR 1.59 (CI 1.14 to 2.24) (*P* = 0.007) but not fall rates (IRR 1.36 (CI 0.95 to 1.97, *P* = 0.09).

Vitamin D levels for each of the seasons are reported in Table 1. There was 15% variation in this variable over the year, with a peak at the end of summer and the lowest values at the end of winter.

Seventy-five percent of fall diaries were posted on schedule while the remaining diaries were returned at the subsequent assessment appointment, resulting in a compliance of 100%. Thirty-three percent of the cohort (29 people) fell at least once, with 10% of the whole group falling multiple times (8 people). Over the duration of the study, 48 falls were recorded: 14 of these occurred inside the house, and 34 occurred outside. Six falls were due to fainting or dizziness and forty due to trip-related events, with one categorised as being pushed over (by a horse) and one not able to be categorised. Twenty-eight falls resulted in injury, with only four requiring medical treatment (including one fracture). Further details on season variation in location and type of fall are provided in Table 2. There were significantly fewer falls during spring than any other season (*P* = 0.01), with no other differences between the seasons recorded. Most falls occurred in May. When falls data were combined from autumn and winter seasons and compared to the combined spring and summer seasons, there were more falls reported in the combined autumn and winter seasons (30 compared to 18). Less injuries from falls were recorded in spring than any other season (*P* = 0.02), with no other seasonal differences recorded.

## 4. Discussion

This is the first cohort study to determine that no seasonal variation of postural sway occurs across 12 months in our population. Significant seasonal variation in serum vitamin D levels, with higher serum levels in summer, was recorded. There was a significant relationship between postural sway and the number of injurious falls observed, with lower values for sway range (i.e., better stability) associated with less fall injuries. The overall fall rates were higher in autumn/winter than spring/summer.

A significant learning effect was seen with all measures of mediolateral postural sway with time point one having larger ranges than all of the other time points (all *P* > 0.05). Further to this, there was no seasonal variation in postural sway under any of the four static balance test conditions measured (eyes open and closed on a firm surface or foam surface). Postural sway range has been used to identify those people with balance impairment [19], and it may be useful in describing the fall-risk status of a particular individual. Our data indicates that postural sway does not appear to be subject to changes across the year within a participant. It has been suggested that this measure is important in describing sensorimotor deficits or disability rather than functional abilities [20] and hence may not be subject to changes that may occur due to altered patterns of activity or sunlight exposure seen seasonally. The stability of this measure in a cohort across a year provides important information for researchers planning interventions designed to impact postural sway and clinicians who are measuring the effectiveness of their interventions. Familiarisation with postural sway measures is recommended. 

Mediolateral sway range has been shown to be an independent fall-risk factor for indoor falls [10]. In our study, a lower proportion of falls occurred indoors (29%) compared to outdoors (71%), and although this is similar to other studies where more healthy samples have been reported as having a greater proportion of falls being outdoor falls (74%; [21]), it may be a reason why no association between sway range and fall incidence was seen. A trend for this association was evident and a larger sample size may have found a significant relationship between these two variables, as this study was powered to determine mediolateral sway changes not fall rates. An association between increased sway range and rate of injurious falls was recorded in our study, reinforcing the importance of this measure for those most at risk of injury.

Overall there was no significant relationship found between postural sway and vitamin D. As increased postural sway is linked to low levels of vitamin D [22], it may be that the levels of vitamin D were sufficient for this parameter even at their lowest levels in participants in this study and did not influence postural sway. If this threshold situation is true, it may be that seasonal variation in postural sway may be present in a population with much lower levels of vitamin D, but this is outside the scope of the current study.

Annual rates of falling for adults over 65 have been reported up to 40% [1], and although our cohort includes some adults between the ages of sixty and sixty-five, with a mean age of 69 years and a fall rate of thirty-three percent, our population appears to be representative of older community-dwelling adults in terms of fall rate. Fall rates in older-old adults (over 75 years) have been shown to vary seasonally [14], but consistent data for the general population of healthy older community-dwelling adults has not been previously reported. One previous study grouped the peak seasons of winter and autumn together and found differences in fall rates [14]. Manipulation of our data in a similar way reveals that there were more falls during the autumn/winter half year compared to spring/summer (Table 2). These seasonal differences may be related to intrinsic factors that may be subject to seasonal variation (e.g., vitamin D, physical activity, and muscle strength) [10] as well as seasonally related environmental factors (e.g., weather, temperature). Further research needs to investigate interventions to address potentially modifiable factors to reduce the increased falls risk in the autumn/winter period. 

Our data indicates a higher rate of falls in summer than has previously been reported [14], perhaps due to activity characteristics of our cohort. Summer and winter falls differ between the genders, with more men falling due to slips in winter and more women falling due to trips in summer [23]. The high proportion of women in our study (79%) may be a factor in the high rate of summer falls observed. Gender differences may also need to be explored in future research in this area [15].

Another factor to consider is the relationship between fall status and vitamin D. Although 60 nmol/L has been determined to be the cutoff for fall-risk function [24], 16/48 falls (33%) occurred in participants in this study whose vitamin D was above that cutoff level. This may be explained by the higher proportion of summer time falls observed in our study. During summer months, with longer hours of daylight at this latitude, a large proportion of the falls occurred in the outside (13 of the 14 falls −93%) (Table 2). By contrast, in winter out-of-doors fall rate was reduced to 64%, indicating a higher winter time proportion of inside falls. For our generally healthy study population, it is likely that our participants were engaged in outdoor activities with higher associated risk of falls during the warmer weather; for example, several summer falls occurred while bushwalking.

Fall injuries, especially fractures, have been found to increase in winter; this includes both inside (hip) and out-of-doors falls (wrist) [25]. Studies in the area of seasonal variation in fracture rates provide good evidence for increased fracture rates from falls in winter, but these appear in populations with older participants than ours (mean age over 75 years) [8, 13]. Our study recorded few injuries that required medical attention, hence making it difficult to compare serious fall injury data.

## 5. Limitations

Although this study aimed to recruit independently living community-dwelling older adults, bias in the sample may be present, as volunteers to this type of research project may be more robust than the community members at large. The lack of more frail subgroups, including those with cognitive impairment, depression, or using walking aids, limits the generalisability of this study. This study provides some evidence that measurements of postural sway may be affected by some effect of test-retest learning, and this needs to be considered in future research.

## 6. Conclusion

This is the first study to investigate the effects of season and postural sway and found that postural sway remained stable over the 12 months. Higher mediolateral sway range was associated with higher rates of injurious falls. No relationship between vitamin D levels was found with postural sway and fall incidence. This study provides important evidence for clinicians and researchers that postural sway remains stable over an annual cycle but may be influenced by a learning effect.

## Figures and Tables

**Table 1:** Variations in serum vitamin D and dynamic and postural balance over 5 seasons.

Variable	End of spring	End of summer	End of autumn	End of winter	End of spring
ML sway EO (cm) mean (SD)	1.9 (0.7)	1.2 (0.6)	1.3 (0.5)	1.1 (0.5)	1.1 (0.5)
Range	0.7–5.0	0.4–3.8	0.6–2.8	0.4–3.6	0.5–2.5
Change from season 1 mean (95% CI)		−0.6 (−0.7 to −0.5)*	−0.6 (−0.7 to −0.4)*	−0.8 (−0.9 to −0.6)*	−0.7 (−0.8 to −0.4)*
ML sway EC (cm) mean (SD)	2.1 (0.9)	1.4 (0.7)	1.4 (0.7)	1.3 (0.8)	1.4 (0.8)
Range	0.8–4.7	0.4–3.8	0.6–4.2	0.6–5.2	0.5–4.9
Change from season 1 mean (95% CI)		−0.7 (−0.8 to −0.6)*	−0.7 (−0.8 to −0.6)*	−0.8 (−0.9 to −0.6)*	−0.7 (−0.9 to −0.6)*
Foam ML (cm) sway EO mean (SD)	3.8 (1.2)	2.8 (1.1)	3.0 (1.2)	2.4 (0.8)	2.6 (0.8)
Range	1.0–8.4	1.1–7.0	1.2–11.41	1.2–6.0	1.1–5.8
Change from season 1 mean (95% CI)		−1.0 (−1.2 to −0.7)*	−0.8 (−1.0 to −0.5)*	−1.2 (−1.5 to −1.0)*	−1.2 (−1.4 to −0.9)*
Foam ML (cm) sway EC mean (SD)	6.8 (2.7)	5.0 (1.7)	4.7 (2.1)	4.6 (1.9)	4.8 (1.9)
Range	2.0–28.7	1.7–10.5	2.0–14.0	2.0–11.9	1.9–16.2
Change from season 1 mean (95% CI)		−1.7 (−2.3 to −1.2)*	−2.1 (−2.6 to −1.5)*	−2.1 (−2.8 to −1.5)*	−2.1 (−2.8 to −1.3)*
Serum vitamin D (nmol/L) mean (SD)	60 (19)	68 (21)*	58 (21)	52 (21)*	59 (20)
Range	20–115	10–126	13–115	16–107	20–114
Change from season 1 mean (95% CI)		8 (6 to 11)	−2 (−5 to 1)	−8 (−11 to −5)	−2 (−5 to 1)

Data presented as mean (SD), changes from season one presented as mean change (95% CI) (adjusted for age, gender strength, and physical activity), *(*P* < 0.05), ML: mediolateral, EO: eyes open, EC: eyes closed, cm: centimeter.

**Table 2:** Fall incidence, injury, location, and cause reported as grouped by season.

Season	Single falls	Multiple falls	Total number of falls	Inside	Outside	Injuries	Trips/slips	Faint
Spring	6	0	6	0	6	3	5	1
Summer	12	0	12	1	11	7	10	2
Autumn	15	2 × 2	19	9	10	12	18	1
Winter	11	0	11	4	7	6	9	2

## Abbreviations

CI: Confidence intervals
EO: Eyes open
EC: Eyes closed
IRR: Incident rate ratio
ML: Mediolateral
RaR: Relative risk
RR: Relative rate
SD: Standard deviation.
